# Mechanistic Insights into the Mechanism of Allosteric Inhibition of Ubiquitin-Specific Protease 7 (USP7)

**DOI:** 10.3390/biom15060749

**Published:** 2025-05-22

**Authors:** Xuebin Wang, Ning Liu, Nuan Li, Shaoyong Lu, Zongtao Chai

**Affiliations:** 1 Department of Pharmacy, Shanghai Children’s Hospital, School of Medicine, Shanghai Jiao Tong University, Shanghai 200062, China; binxuewang@sjtu.edu.cn; 2 Key Laboratory of Protection, Development and Utilization of Medicinal Resources in Liupanshan Area, Ministry of Education, Peptide & Protein Drug Research Center, School of Pharmacy, Ningxia Medical University, Yinchuan 750004, China; 20160126@nxmu.edu.cn; 3 Medicinal Chemistry and Bioinformatics Center, Shanghai Jiao Tong University School of Medicine, Shanghai 200025, China; linuan123@sjtu.edu.cn; 4 Department of Hepatic Surgery, Shanghai Geriatric Medical Center, Shanghai 201104, China; 5 Department of Liver Surgery and Transplantation, Liver Cancer Institute and Zhongshan Hospital, Fudan University, Shanghai 200032, China

**Keywords:** molecular dynamics simulations, conformational dynamics, allosteric inhibition, protein–protein interactions, allosteric communication

## Abstract

Ubiquitin-specific protease 7 (USP7), a deubiquitinase enzyme responsible for removing ubiquitin (Ub) from target proteins, plays a crucial role in oncogenic pathways and has been implicated in various human diseases. X-ray crystallography has revealed distinct conformations of USP7, including apo (ligand-free), allosteric inhibitor-, and Ub-bound states. However, the dynamic mechanisms underlying the allosteric inhibition of USP7 remain unclear. This study investigates the effect of allosteric inhibitor binding on the dynamics of USP7 through multiple replica molecular dynamics simulations. Our results demonstrate that Ub binding stabilizes the USP7 conformation, while allosteric inhibitor binding increases flexibility and variability in the fingers and palm domains of USP7. Furthermore, our analysis of USP7 local regions reveals that allosteric inhibitor binding not only restrains the dynamics of the C-terminal Ub binding site, thereby impeding the accessibility of Ub to USP7, but also disrupts the proper alignment of the catalytic triad (Cys223-His464-Asp481) in USP7. Additionally, community network analysis indicates that intra-domain communications within the fingers domain in USP7 are significantly enhanced upon allosteric inhibitor binding. This study reveals that the binding of an allosteric inhibitor induces a dynamic shift in enzyme’s conformational equilibrium, effectively disrupting its catalytic activity through allosteric modulation.

## 1. Introduction

The ubiquitin-proteasome system (UPS), a highly conserved eukaryotic protein quality control mechanism, governs intracellular proteostasis through targeted substrate degradation [1]. This system operates via a coordinated enzymatic cascade involving ubiquitin-activating enzymes (E1), ubiquitin-conjugating enzymes (E2), ubiquitin ligases (E3), deubiquitinases (DUBs), ubiquitin (Ub), and the 26S proteasome complex [2]. Central to UPS functionality is ubiquitination—a dynamic enzymatic process wherein Ub molecules are covalently linked to specific protein substrates. This post-translational modification is sequentially mediated by the E1-E2-E3 enzymatic triad, ultimately marking substrates for proteasomal recognition and degradation. Conversely, deubiquitination, catalyzed by DUBs, removes Ub from target proteins, reversing the ubiquitination process. These processes regulate protein stability, function, and localization [1].

**Figure 1:**
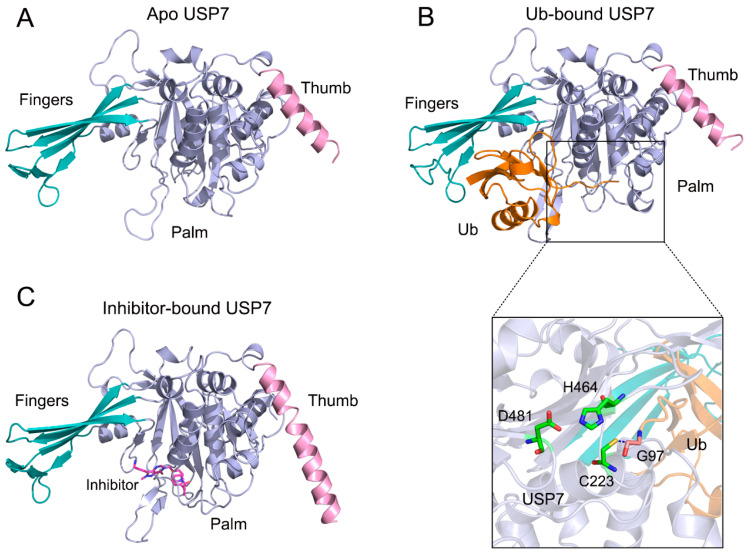
(**A**) Molecular architecture of apo USP7 (PDB entry 1NB8), with domain color-coding: figures (pink), palm (cyan), and thumb (light blue) subdomains. (**B**) Ub-bound USP7 (PDB entry 1NBF) illustrating induced fit mechanism. Ub (orange surface) engages the fingers-palm interface, while structural magnification reveals catalytic triad reconfiguration (Cys223/His464/Asp481) and thioester bond formation between USP7 Cys223 sulfur atom and Ub Gly97 carboxyl oxygen. (**C**) Allosteric inhibition complex (PDB entry 5N9T) showing compound **5** (stick model) binding distal to the catalytic cleft. Secondary structure elements maintain the standard coloring scheme across panels.

There are ~100 DUBs evolutionarily grouped into two major classes based on their enzymatic mechanism: cysteine proteases and metalloproteases. Among these, ubiquitin-specific protease 7 (USP7), a cysteine protease extensively documented in studies [3,4,5,6,7,8], exemplifies structural complexity through its multi-domain architecture. This 1102-residue enzyme contains four functionally specialized regions: (i) an N-terminal disordered segment (residues 1–50), featuring a polyglutamine (Poly-Q) repeat, (ii) a TRAF-homology domain (residues 53–205) mediating protein interactions, (iii) a central catalytic core (residues 208–560) responsible for Ub recognition and isopeptidase activity, and (iv) a C-terminal quintuple UBL domain array (residues 564–1102) modulating substrate specificity [9,10,11]. Architecturally, the catalytic domain adopts a tripartite papain-like fold—comprising fingers, palm, and thumb subdomains (Figure 1A)—a structural blueprint conserved across USP family members [12,13]. Mechanistic crystallographic analyses reveal that Ub binding within the fingers–palm interface induces conformational restructuring of the catalytic triad (Cys223/His464/Asp481), transitioning these residues into catalytically competent orientations essential for efficient Ub chain hydrolysis (Figure 1B). Crucially, this domain serves dual roles: direct Ub engagement and spatial coordination of substrate deubiquitination events.

Recently, a small-molecule inhibitor (compound 4) was reported to exhibit high affinity and specificity for USP7 (IC_50_ = 6 ± 2 nM) both in vitro and in human cells [14]. The X-ray co-crystal structure of the catalytic domain of USP7 in complex with compound 5, a close analog of compound 4, reveals occupancy of a non-canonical allosteric pocket within the palm subdomain (Figure 1C), distinct from Ub-binding regions. Despite minimal global structural perturbations compared to apo USP7 (Cα RMSD 0.38 Å), the inhibitor-bound state exhibits marked divergence from Ub-complexed conformations (Cα RMSD 0.79 Å), suggesting ligand-specific domain reconfigurations. Moreover, allosteric inhibitor binding may misalign the catalytic triad, leading to a catalytically non-productive conformation. While these existing structural snapshots (apo, Ub-bound, inhibitor-bound) provide useful information about Ub and inhibitor interaction with the enzyme, the underlying dynamic mechanism regarding the allosteric inhibition of USP7 catalytic domain remains to be fully elucidated because the overall conformational dynamics of USP7 catalytic domain are rarely captured by structural methods.

All-atom molecular dynamics (MD) simulations offer atomically detailed information on protein conformational dynamics, sampling multiple timescales from femtoseconds to microseconds to reach biologically relevant timescales (microseconds and beyond), such as signal transduction, enzyme catalysis, allosteric transitions, and protein-ligand or protein-protein binding [15,16,17]. Thus, MD simulations can complement experiments by providing atomic-level detail necessary for monitoring the progression of conformational changes in proteins [18,19,20,21,22,23,24]. To address the mechanism of allosteric inhibition of USP7 catalytic domain, we employed multi-replica MD simulations to sample three functional states (apo, Ub-bound, inhibitor-bound). An integrated conformational analysis method, including dynamic cross-correlation matrix analysis and network analysis, was used to reveal state-specific dynamic signatures. The simulation-derived conformational landscapes provide critical insights for allosteric inhibitor design.

## 2. Materials and Methods

### 2.1. System Preparation

The starting coordinates were obtained from the Protein Data Bank (www.rcsb.org) for three systems: apo USP7 (PDB entry 1NB8) [25], USP7 in complex with Ub (PDB entry 1NBF) [25], and USP7 bound to compound 5 (PDB entry 5N9T) [14]. Structural analysis revealed that in the USP7-Ub complex, the catalytic cysteine residue (Cys223) of USP7 establishes a covalent linkage with the C-terminal glycine (Gly97) of Ub. For modeling purposes, the (*R*)-trifluoromethyl moiety in compound 5 was computationally modified to an (*R*)-methyl group to represent compound 4. Any incomplete side chain coordinates were reconstructed using UCSF Chimera’s modeling tools [26].

### 2.2. MD Simulations

All MD simulations were executed using the Amber (Version: 18) 18 software package [27]. Hydrogen atoms were added to three simulated systems (apo USP7, USP7-Ub complex, and USP7-compound 4 complex) using the *tleap* module. Force field assignments included the Amber ff14SB for protein residues [28] and the General Amber Force Field (GAFF) for the small-molecule inhibitor (compound 4) [29]. The covalent bond between USP7 Cys223 and the C-terminal carboxyl group of Ub’s Gly97 was explicitly defined in *tleap*. Compound 4 underwent geometry optimization using the semi-empirical AM1 method, followed by bond-charge correction (BCC) charge assignment via the *Antechamber* module [30]. Protonation states of amino acid residues were determined at physiological pH using PROPKA (Version: 3.0) [31]. Each system was solvated in a truncated octahedron periodic box filled with TIP3P water [32], maintaining a minimum 10 Å water layer around the protein surface, and neutralized with Na^+^ counterions.

Energy minimization involved two phases: 20,000 cycles with positional restraints on protein atoms and 50,000 cycles of unrestrained minimization. Systems were then gradually heated from 0 K to 300 K over 100 ps under constant volume (NVT ensemble), followed by 200 ps equilibration. Production simulations were conducted in the NPT ensemble (300 K, 1 atm) using three independent 1000 ns trajectories initialized with random velocities. Electrostatic interactions were computed using the Particle Mesh Ewald (PME) method [33], and hydrogen-containing bonds were constrained via the SHAKE algorithm [34]. A 2 fs integration time step was employed.

All simulations utilized the *pmemd.cuda* GPU-accelerated module in Amber 18, with temperature and pressure regulation achieved through the Langevin thermostat/barostat (1.0 ps coupling time) [35]. This protocol ensured reproducible trajectories for subsequent structural and dynamical analyses.

### 2.3. DCCM Analysis

To characterize residue-level dynamical coupling, cross-correlation coefficients (C_ij_) between Cα atoms were computed using trajectory-averaged calculations. The normalized covariance metric was defined as [36,37,38,39]:

$$C\left(i,j\right)=\frac{\langle \Delta r_{i}\cdot \Delta r_{j}\rangle }{\sqrt{<\Delta r_{i}{>}^{2}\cdot <\Delta r_{j}{>}^{2}}}$$

where Δri (Δrj) denotes the displacement vector of residue *i* (*j*) relative to its equilibrium position, and angle brackets represent averaging across concatenated replica trajectories.

### 2.4. Community Network Analysis

The residue interactions network of USP7 was generated from the DCCM through NetworkView [40,41], a Tcl plugin integrated with VMD [42]. In this representation, each residue’s Cα atom served as a network node, with edges established between node pairs maintaining spatial proximity (≤4.5 Å) for over 75% of the simulation duration [43,44]. Edge weights were determined by the logarithmic transformation:

d_i,j_ = −*log*(|C_i,j_|)

where Cij values originated from the Cα-based cross-correlation analysis.

## 3. Results and Discussion

### 3.1. Allosteric Modulation Amplifies USP7 Structural Plasticity

**Figure 2:**
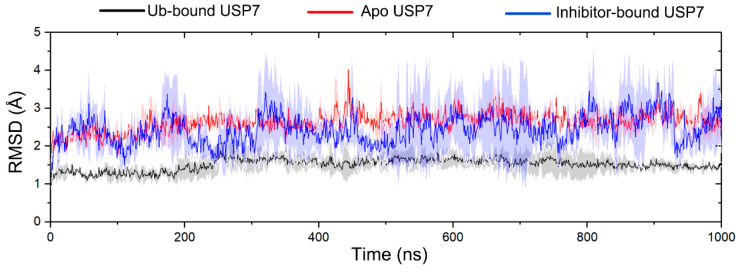
The root-mean-square deviation (RMSD) of Cα atoms for USP7 in the Ub-bound (black line), apo (red line), and inhibitor-bound (blue line) states along the 1000 ns MD simulations. The shaded region denotes variability (standard deviation) across three independent MD replicates.

To comprehensively investigate the conformational sampling of the USP7 catalytic domain (residues 208–560, hereafter referred to as USP7) in its apo, inhibitor-bound (compound 4), and Ub-bound states, we implemented MD simulations across three functional states. Each simulation comprised three replicates, with each replicate running for 1 microsecond (μs), resulting in a total simulation time of 3 μs per system. To reveal the convergence and structural stability of USP7 across different states through multiple replicate MD simulations, the Cα RMSD was monitored. MD simulations of all systems generally achieved conformational equilibrium after approximately 300 ns (Figure 2). Consequently, the equilibrium trajectories from 300 to 1000 ns were combined from the three replicates, yielding a single consolidated MD trajectory of 2100 ns for post-processing analyses.

The RMSD values for USP7 in the Ub-bound, apo, and inhibitor-bound states were 1.51 ± 0.23, 2.64 ± 0.25, and 2.45 ± 0.66 Å for the USP7, respectively. These results indicated that Ub binding significantly contributes to the stabilization of conformational plasticity of USP7, whereas the inhibitor-bound enzyme retains considerable flexibility, similar to the apo form.

**Figure 3:**
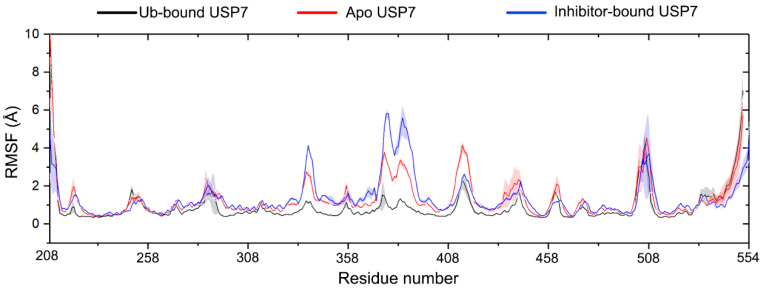
Root mean square fluctuation (RMSF) profiles of Cα atoms are shown for Ub-bound (black), apo (red), and inhibitor-bound (blue) USP7. Shaded regions reflect statistical variability across triplicate MD simulations (standard deviation).

Quantitative residue-wise dynamics were characterized through Cα-root mean square fluctuation (Cα-RMSF) profiling. While it is indeed commonly observed that inhibitor-bound protein complexes exhibit reduced conformational flexibility compared to apo forms, our MD simulations revealed a striking exception in USP7. The RMSF profiling demonstrated preserved global conformational sampling capacity in USP7 between apo and inhibitor-engaged states (Figure 3), with the most remarkable fluctuations observed in the fingers domain (residues 333–348 and 370–400). Comparing to apo USP7, inhibitor binding notably increases the structural flexibility of the fingers domain. The comparable RMSF values between apo USP7 and inhibitor-bound USP7 suggest a unique allosteric regulation mechanism: the catalytic domain maintains intrinsic flexibility even when occupied by inhibitors. These findings challenge the paradigm of ligand-induced rigidity and offer new insights for developing allosteric inhibitors targeting flexible enzymes. Conversely, Ub binding markedly stabilizes this region due to direct interactions between Ub and the fingers domain of USP7.

We next compared the NMR data of the USP7 catalytic domain in both its unbound state and in complex with Ub. Ub interaction elicited pronounced chemical shift perturbations localized to the palm subdomain of USP7, as documented in prior work [9], indicating a stable palm-Ub binding interface upon Ub binding. Consistent with these experimental observations, our RMSF analysis of the apo USP7 catalytic domain versus the USP7 catalytic domain-Ub complex revealed that, in the complex system, the USP7 catalytic domain exhibited reduced RMSF.

### 3.2. Allosteric Inhibitor Binding Enhances the Internal Motions of USP7

To elucidate the influence of allosteric inhibitor interactions on USP7 conformational dynamics, Cα-based dynamic cross-correlation matrices (DCCM) were generated using the CPPTRAJ module in Amber 18 [45,46,47]. The color-coded modes are utilized to display the degree of correlated or anti-correlated motions between residues. Orange represents the correlated motions (C_ij_ > 0) between specific residues, while magenta indicates the anti-correlated motions (C_ij_ < 0) between residues. Weak pairwise couplings (|C_ij_| < 0.4) were filtered as white space in DCCM visualizations, reflecting statistically insignificant inter-residue motion coordination.

**Figure 4:**
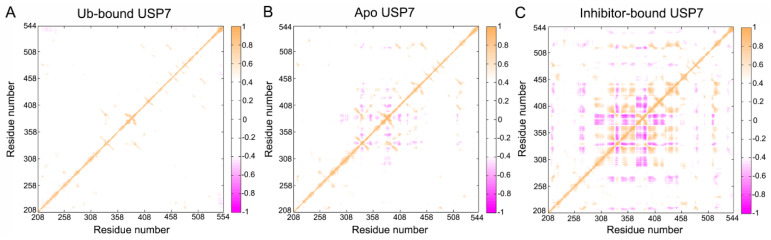
Dynamic coordination landscapes derived from Cα atom trajectories demonstrate conformational coupling differences between: (**A**) Ub-bound, (**B**) apo, and (**C**) inhibitor-bound USP7 states. A bidirectional color scheme (–1 ≤ C_ij_ ≤ 1) quantifies residue pair correlations, with white masking coefficients |C_ij_| < 0.4 following statistical validation through trajectory subsampling.

As shown in Figure 4, allosteric inhibitor binding significantly alters the movement pattern of USP7. For Ub-bound USP7 (Figure 4A), there is minimal intradomain motion, indicating that Ub binding strongly stabilizes the conformational flexibility of USP7. For apo USP7 (Figure 4B), significant anti-correlated motions are observed between residues 320 and 440, which belong to the fingers domain. In contrast, allosteric inhibitor binding strengthens both correlated and anti-correlated motions within residues 320–440 (fingers domain). In addition, allosteric inhibitor binding slightly increases anti-correlated motions between residues 260 and 270 (palm domain) and residues 320 and 440 (fingers domain), as well as correlated motions between residues 505 and 515 (palm domain) and residues 320 and440 (fingers domain). Notably, the changes in RMSF values in the inhibitor-bound USP7, particularly in the fingers domain (residues 333–348 and 370–400) and palm domain (residues 505–515), align with the DCCM findings.

### 3.3. Allosteric Inhibitor Binding Restricts Ub Accessibility to USP7

**Figure 5:**
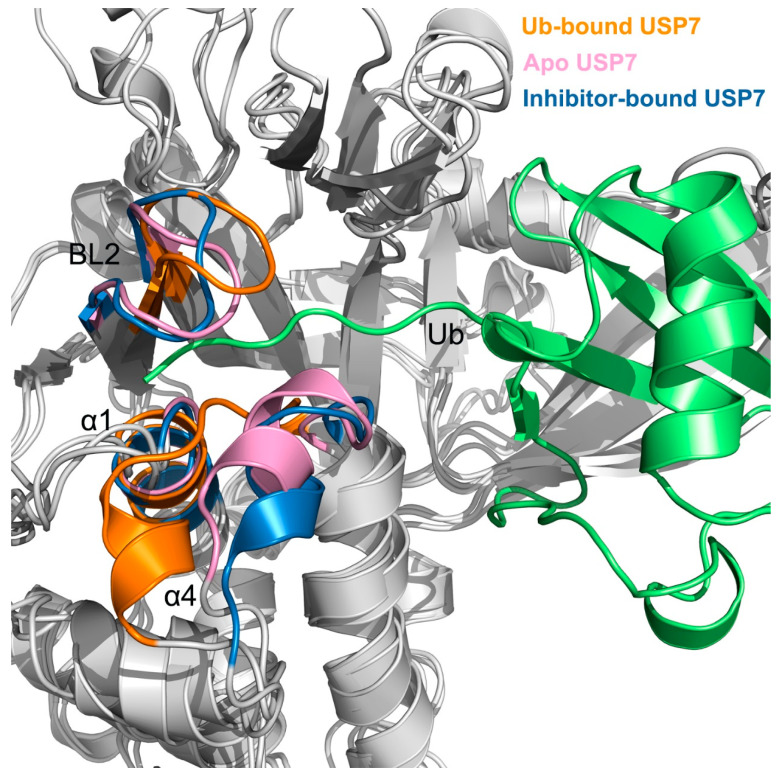
Superimposition of the USP7 backbone reveals distinct conformational shifts in key structural regions, specially the BL2 loop and helices α1 and α4 across its Ub-bound (orange), apo (pink), and inhibitor-bound (blue) states. The Ub is depicted in green.

Structural analysis of the USP7-Ub interface identifies three critical recognition elements: α1/α4 helical domains and the β-hairpin BL2 loop (residues 456–464), which cooperatively stabilize the Ub C-terminus. To quantify allosteric perturbations induced by inhibitor binding, representative structural complexes of USP7 in different states were obtained using cluster analysis of MD trajectories [48]. Figure 5 illustrates the backbone superimposition of USP7 across apo, inhibitor-bound, and Ub-bound states. Notably, the conformations of helix α4 and the BL2 loop are similar between these ligand-free and inhibitor-complexed conformations, but differ significantly from those in the Ub-bound state. Specially, both the ligand-free and inhibitor-bound states exhibit a “down” conformation of the BL2 loop and an “outward” conformation of helix α4, while the Ub-bound state shows an “up” conformation of the BL2 loop and an “inward” conformation of helix α4. Consequently, the pronounced conformational changes in the BL2 loop and helix α4 upon allosteric inhibitor binding may lead to a contraction of the C-terminal Ub binding site, thereby restraining the accessibility of Ub to the USP7.

**Figure 6:**
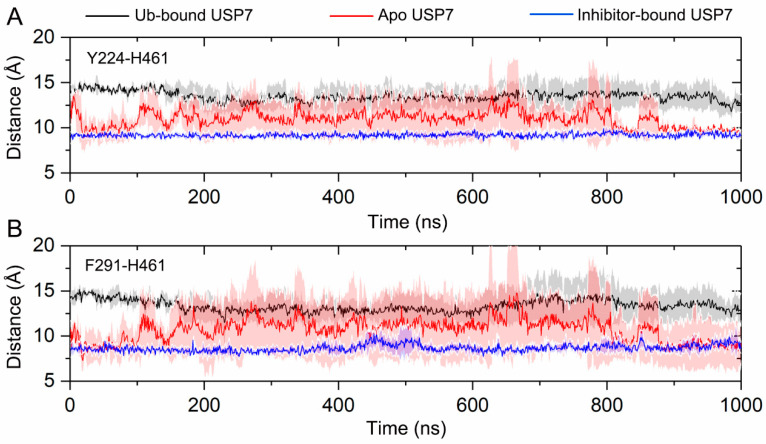
(**A**) The Cα-Cα distance between Tyr224 (helix α1) and His461 (BL2 loop) for the Ub-bound (black line), apo (red line), and inhibitor-bound (blue line) USP7 along the 1000 ns MD simulations. (**B**) The Cα-Cα distance between Phe291 (helix α4) and His461 (BL2 loop) for the Ub-bound (black line), apo (red line), and inhibitor-bound (blue line) USP7 along the 1000 ns MD simulations. The shaded region denotes variability (standard deviation) across three independent MD replicates.

To test the hypothesis that allosteric inhibitor binding disturbs the Ub binding site in USP7, two distances defining key features of the C-terminal Ub binding site were calculated. Two key interatomic distances were analyzed to quantify conformational changes: the Cα-Cα distance between Tyr224 (located in helix α1) and His461 (within the BL2 loop), and the Cα-Cα distance between Phe291 (positioned in helix α4) and His461 (BL2 loop). These metrics were selected to characterize the overall conformational dynamics of the C-terminal Ub binding site. As shown in Figure 6A, in the Ub-bound USP7, the distance between Tyr224 and His461 remains stable along the simulations, with the value of 13.47 ± 1.35 Å. In the inhibitor-bound USP7, the distance stabilizes at 9.17 ± 0.33 Å throughout the simulations. In contrast, in the apo USP7, the distance fluctuates between values observed in the inhibitor-bound and Ub-bound states, averaging 10.93 Å. Similarly, as depicted in Figure 6B, the distance between Phe291 and His461 remains stable at 13.35 ± 1.68 Å in the Ub-bound USP7 and at 8.66 ± 0.56 Å in the inhibitor-bound USP7. However, in the apo USP7, the distance fluctuates between these two values, averaging 10.72 Å.

**Figure 7:**
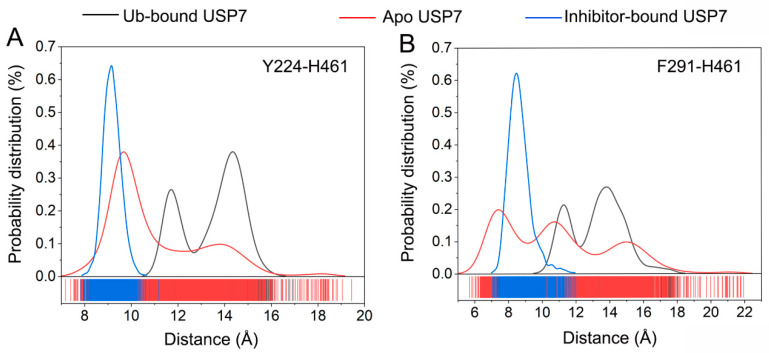
(**A**) Statistical distribution of distances between Tyr224 and His461 Cα atoms for the Ub-bound (black line), apo (red line), and inhibitor-bound (blue line) USP7. (**B**) Statistical distribution of distances between Phe291 and His461 Cα atoms for the Ub-bound (black line), apo (red line), and inhibitor-bound (blue line) USP7.

To probe how allosteric inhibitors modulate USP7’s functional dynamics, we statistically analyzed the conformational sampling of two geometric parameters associated with the C-terminal Ub-binding domain. Figure 7A visualizes the statistical distribution of distances between Tyr224 and His461 Cα atoms across simulations. In the Ub-bound USP7, the distribution exhibits two peaks at 11.5 and 14.5 Å. In the inhibitor-bound USP7, the distribution has a predominant peak at 9.1 Å. In the apo USP7, the distribution shows two peaks, with a major peak at 9.7 Å and a minor peak at 14.0 Å. Figure 7B presents the statistical distribution for distances between Phe291 and His461 Cα atoms. In the Ub-bound USP7, the distribution has two peaks at 11.1 and 13.8 Å. In the inhibitor-bound USP7, the distribution has a predominant peak at 8.7 Å. In the apo USP7, the distribution exhibits three peaks at 7.2, 10.8, and 15.1 Å. Taken together, these results suggest that allosteric inhibitor binding leads to a closed C-terminal Ub binding site, thereby impeding Ub’s spatial entry to USP7’s catalytic interface. Conversely, MD trajectories of apo USP7 demonstrate conformational plasticity in the C-terminal domain, facilitating Ub binding.

### 3.4. Allosteric Inhibitor Binding Disrupts the Catalytic Triad Arrangement

**Figure 8:**
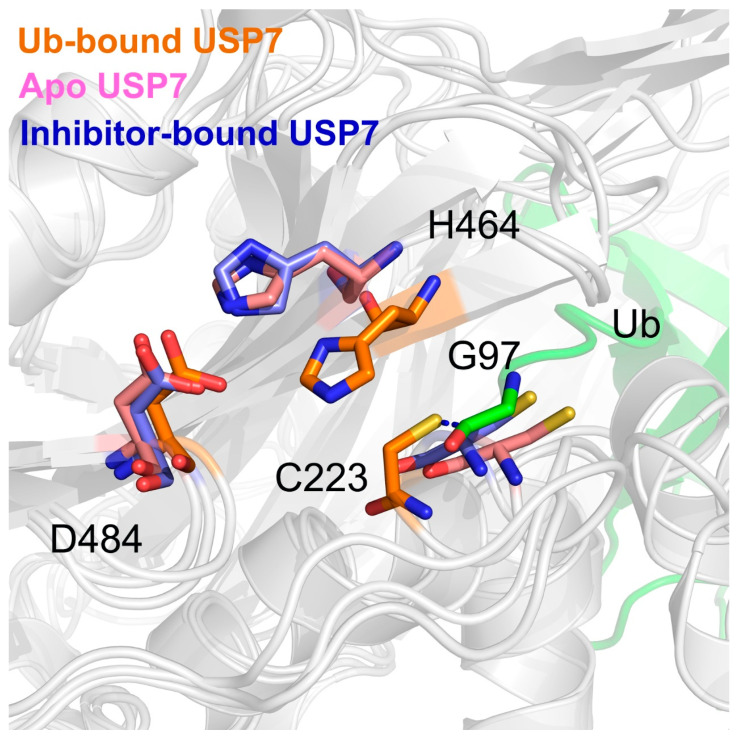
The overlap of USP7’s catalytic triad (Cys223, His464, Asp481) in the Ub-bound (orange), apo (pink), and inhibitor-bound (blue) states. The Gly97 at the C-terminal Ub is showing in green.

To resolve conformational perturbations in USP7’s catalytic triad (Cys223, His464, Asp481) induced by allosteric regulation, we performed structural alignment of three distinct complexes: USP7-Ub holoenzyme, inhibitor-bound USP7, and apo USP7 (Figure 8). Structural analysis of the USP7-Ub complex reveals precise catalytic triad coordination essential for enzymatic competence. The nucleophilic thiol of Cys223 engages Ub’s C-terminal Gly97 through thioester bond formation, while His464 maintains catalytic geometry by simultaneously coordinating the Cys223 sulfhydryl group and Asp481 carboxylate moiety. However, both the inhibitor-bound and apo USP7 exhibit a misaligned catalytic triad, with significant conformational changes in catalytic Cys223 and His464, adopting novel conformations not observed in the Ub-bound USP7. These data indicated that allosteric inhibitor binding may disrupt the proper alignment of the catalytic triad.

**Figure 9:**
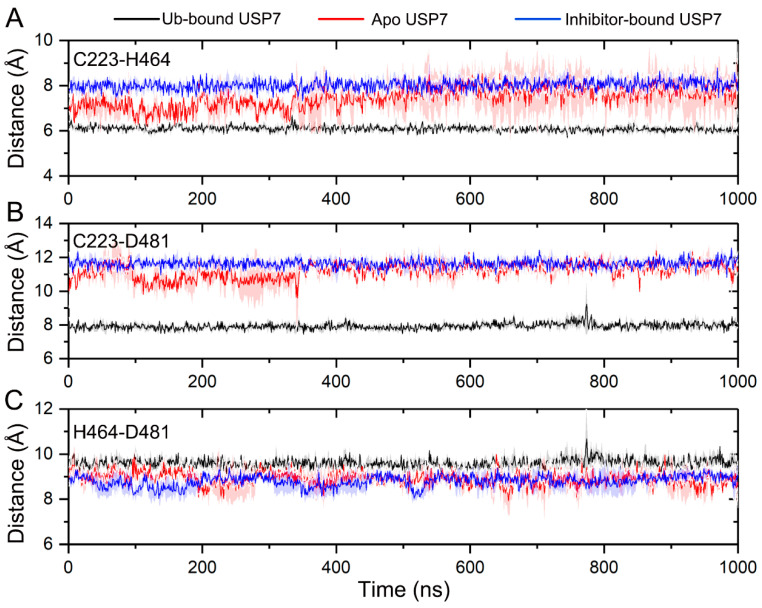
The Cα-Cα distances between Cys223 and His464 (**A**), between Cys223 and Asp481 (**B**), and between His464 and Asp481 (**C**) for the Ub-bound (black line), apo (red line), and inhibitor-bound (blue line) USP7 along the 1000 ns MD simulations. The shaded region denotes variability (standard deviation) across three independent MD replicates.

To test the hypothesis that allosteric inhibitor binding leads to a misaligned catalytic triad, the inter-residue distances among Cys223, His464, and Asp481 were calculated. As illustrated in Figure 9A, the Cα-Cα distance between Cys223 and His464 maintains stability throughout the simulations, with values of 6.09 ± 0.17 Å for Ub-bound USP7 and 8.02 ± 0.32 Å for inhibitor-bound USP7. In contrast, the distance fluctuates in apo USP7 during the 0–350 ns simulation, averaging 7.08 Å and reaching up to 7.7 Å in subsequent MD simulations. For the inter-residue distance between the two Cα atoms of Cys223 and Asp481 (Figure 9B), stability is observed across all three simulated systems, with respective values of 7.93 ± 0.25 Å for Ub-bound USP7, 11.62 ± 0.36 Å for inhibitor-bound USP7, and 11.20 ± 0.55 Å for apo USP7. The inter-residue distance between the two Cα atoms of His464 and Asp481 shows no appreciable difference among the three simulated systems (Figure 9C), with values of 9.59 ± 0.31 Å for Ub-bound USP7, 8.80 ± 0.37 Å for inhibitor-bound USP7, and 8.90 ± 0.41 Å for apo USP7.

**Figure 10:**
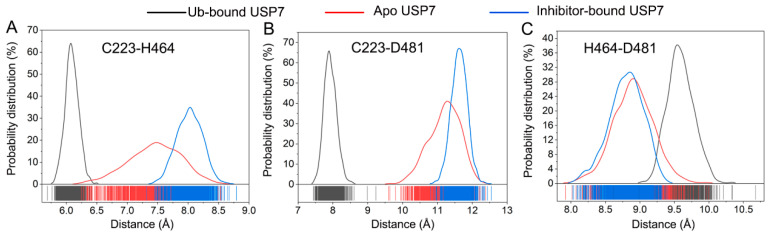
Statistical distributions of the Cα-Cα distance between Cys223 and His464 (**A**), between Cys223 and Asp481(**B**), and between His464 and Asp481 (**C**) for the Ub-bound (black line), apo (red line), and inhibitor-bound (blue line) USP7.

Quantitative assessment of catalytic triad dynamics was performed through conformational sampling analysis. Figure 10A presents the statistical distribution for the Cα-Cα distance between Cys223 and His464. The distribution displays a predominant peak for all three simulated systems, with a peak at 6.1 Å for Ub-bound USP7, 8.1 Å for inhibitor-bound USP7, and 7.4 Å for apo USP7. Similarly, the probability distribution for the distance between the two Cα atoms of Cys223 and Asp481 also exhibits a predominant peak for all three simulated systems (Figure 10B), with a peak at 7.9 Å for Ub-bound USP7, 11.6 Å for inhibitor-bound USP7, and 11.1 Å for apo USP7. For the probability distribution for the Cα-Cα distance between H464 and Asp481 (Figure 10C), a single predominant peak is observed at 9.6 Å for Ub-bound USP7, 8.8 Å for inhibitor-bound USP7, and 8.9 Å for apo USP7. Cumulatively, the calculated inter-residue distances and their probability distributions suggested that allosteric inhibitor binding disrupts the proper alignment of the catalytic triad.

### 3.5. Topological Reorganization of Allosteric Networks

**Figure 11:**
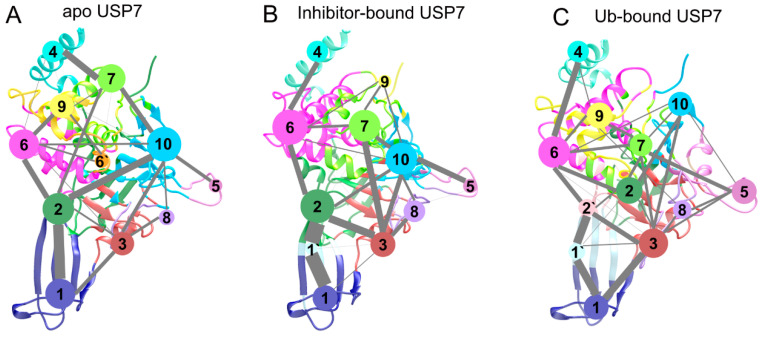
Community networks of USP7. States include: (**A**) apo, (**B**) inhibitor-bound, and (**C**) Ub-bound configurations. Edge thickness encodes inter-community contact persistence.

To map ligand-induced perturbations in USP7’s signal propagation pathways, we conducted community network analysis (CNA) to evaluate dynamic networks in three different states. CNA is a robust method for exploring conformational changes in proteins by decoupling correlated motions between residues [49,50,51,52]. If a cluster of residues have the same movements, they are classified into the same community. Two communities are connected by an edge whose thickness means the intensity of the community coupling between them [53,54]. Figure 11 illustrates the 3D representations of community networks of apo, Ub-bound, and inhibitor-bound USP7. In general, the community networks in apo (Figure 11A) and inhibitor-bound (Figure 11B) USP7 are similar, while those in Ub-bound USP7 exhibit alterations (Figure 11C).

In both the apo and inhibitor-bound USP7, all residues were classified into 11 communities, whereas Ub-bound USP7 had 12 communities. Specially, in apo USP7, communities 1, 2, and 6 encompass the fingers domain, representing the binding site for Ub. Upon inhibitor binding, community 1 in the apo state splits into two communities (1 and 1’). Furthermore, in inhibitor-bound USP7, communities 1 and 2 from the apo state divide into four communities (1, 1’, 2 and 2’). Compared to apo USP7, allosteric inhibitor binding strengthens the coupling between communities 1 and 2 via community 1’. Conversely, in Ub-bound USP7, communication among communities 1, 1’, 2, and 2 weakens upon Ub binding compared to the communication between communities 1 and 2 in apo USP7 among communities 1, 1’, and 2 in inhibitor-bound USP7. These results indicate that allosteric inhibitor binding enhances intra-domain coupling with the fingers domain of USP7, suggesting increased flexibility consistent with RMSD and RMSF results. This enhanced flexibility may hinder the binding of Ub to the catalytic site, thus inactivating USP7.

## 4. Conclusions

Given the multifaced nature of USP7 in disease, it is becoming an increasingly attractive target for cancer treatment. Accurately elucidating the allosteric inhibition mechanism of USP7 is crucial for gaining deeper insights into its biological function. In this study, all-atom MD simulations totaling 3 μs of simulation time, comprising three independent 1 μs simulations, were carried out on apo, inhibitor-, and Ub-bound USP7 to enhance conformational sampling and explore the allosteric mechanism of USP7 by the inhibitor.

Through analyses, such as RMSF analysis, cross-correlation, network mapping, and detailed evaluations of USP7-inhibitor and USP7-Ub interactions, we gained valuable insights into the allosteric inhibition mechanism mediated by the inhibitor. The results suggest that allosteric inhibitor binding increases the conformational flexibility of USP7 and markedly impacts the intradomain movements of the fingers domain (residues 320–440) and palm domain (residues 260–270 and residues 505–515). Structurally, the fingers domain constitutes the binding site for Ub; therefore, alterations in flexibility, motion patterns, and dynamic behavior of USP7 contribute to its allosteric inhibition. Analysis of the C-terminal Ub binding site and the arrangement of the catalytic triad indicates that allosteric inhibitor binding leads to closure of the C-terminal Ub binding site, thereby restraining Ub accessibility to USP7. This also disrupts the proper alignment of catalytic triad (Cys223-His464-Asp481) within USP7. Additionally, network community analysis suggests that allosteric inhibitor binding strengthens intra-domain communications within the fingers domain of USP7, potentially hindering Ub binding to USP7.

While the present investigation centers on the USP7 catalytic domain, subsequent research will employ integrative structural modeling approaches to develop a full-length USP7-allosteric inhibitor complex. This future work will leverage the catalytic domain-inhibitor architecture established in our current study to elucidate the structural basis of allosteric regulation across the entire enzyme.

## Data Availability

Data are contained within the article.
